# Changes in the Healthy Beverage Index in Response to an Intervention Targeting a Reduction in Sugar-Sweetened Beverage Consumption as Compared to an Intervention Targeting Improvements in Physical Activity: Results from the Talking Health Trial

**DOI:** 10.3390/nu7125525

**Published:** 2015-12-04

**Authors:** Valisa E. Hedrick, Brenda M. Davy, Emily A. Myers, Wen You, Jamie M. Zoellner

**Affiliations:** 1 Department of Human Nutrition, Foods, and Exercise, Virginia Polytechnic Institute and State University, 295 West Campus Drive, Blacksburg, VA 24061, USA; bdavy@vt.edu (B.M.D.); eamyers@vt.edu (E.A.M.); zoellner@vt.edu (J.M.Z.); 2 Department of Agricultural and Applied Economics, Virginia Polytechnic Institute and State University, 250 Drillfield Drive, Blacksburg, VA 24061, USA; wenyou@vt.edu

**Keywords:** beverage consumption, sugar-sweetened beverages, beverage quality, dietary quality, Healthy Beverage Index, Healthy Eating Index

## Abstract

The recently developed Healthy Beverage Index (HBI) was designed to evaluate overall beverage intake quality (including total fluid consumption and beverage calories), yet no known intervention studies have assessed longitudinal changes to the HBI. The objective of this investigation was to assess changes in HBI scores in response to a sugar-sweetened beverage (SSB) reduction trial as compared to a physical activity comparison group. Participants were enrolled into a six-month, community-based, controlled behavioral trial and randomized into either a SSB reduction group (SIPsmartER) or a physical activity group (MoveMore). Correlations and multilevel mixed-effects linear regression with intention-to-treat analyses are presented. Total HBI score significantly increased for SIPsmartER (*n* = 149) (mean increase = 7.5 points (5.4, 9.7), *p* ≤ 0.001) and MoveMore (*n* = 143) (mean increase = 3.4 points (1.6, 5.2), *p* ≤ 0.001) participants, with a significant between group effect (*p* ≤ 0.05), over the six-month intervention. Other significant changes in HBI components for SIPsmartER included increased SSB and total beverage calorie scores, and decreased low-fat milk and diet soda scores. Changes in total HBI scores were significantly correlated with changes in total Healthy Eating Index-2010 scores (*r* = 0.15, *p* ≤ 0.01). Our findings suggest that individual HBI component scores, beyond the SSB component, are influenced by intervention strategies that primarily focus on SSB reduction.

## 1. Introduction

Extensive research has investigated changes in beverage consumption, especially sugar-sweetened beverages (SSB) [1,2], due to the health consequences associated with excessive intake, obesity [3,4,5], diabetes [4,6,7,8], and cardiovascular disease [5]. However, few studies have evaluated the overall quality of beverage consumption within the context of evaluating all consumed beverages as a pattern [9]. Recent studies indicate that adults in the United States consume on average more than 11 teaspoons of added sugar from SSB per day, and yet average water consumption only totals approximately one liter per day, well below the recommended intake of 2.7–3.7 liters per day [10,11,12]. Beverage consumption also impacts total caloric consumption, with total energy intake from all foods and beverages being 7.8% higher when SSB are consumed instead of water [13]. The Scientific Report of the 2015 Dietary Guidelines Advisory Committee suggests that a broader focus beyond SSB is needed and efforts should be concentrated on healthier beverage patterns, rather than on individual beverages [14].

An established method of assessing dietary quality is the Healthy Eating Index (HEI-2010), which is comprised of twelve food groups/categories [15]. The HEI-2010 is not without limitations, especially when attempting to assess beverage intake quality, as it does not take into account the consumption of water, unsweetened tea and coffee, and diet beverages, nor total beverage energy, or total fluid needs. Additionally, 100% fruit juice is included in the total fruit category, and all dairy products (whole and low/fat-free) are grouped in the same category (milk, cheese, yogurt, ice cream, *etc.*). These limitations inhibit the ability of the HEI-2010 to assess beverage intake quality, and its relation to overall dietary quality and health.

Our group recently developed the Healthy Beverage Index (HBI), which includes eight beverage categories as well as total beverage energy and fluid consumption components. The HBI was designed to overcome the limitations of the HEI-2010 by assessing overall beverage quality, in relation to total daily energy and fluid needs, against standards set by the Dietary Guidelines for Americans and the Beverage Guidance Panel [16]. The Beverage Guidance Panel’s recommendations are based on measured fluid intake (fl oz). The majority of HBI components were derived from the Beverage Guidance Panel; however, the recommendations were converted to fluid needs as a percent of total fluid requirements. Additionally, the “caloric beverages with some nutrients” was divided into three categories on the HBI: whole fat milk, 100% fruit juice, and alcohol. To determine total fluid needs, the standard fluid requirement of one mL per kcal consumed was used [16]. Further detail is provided in Table 1. As with the HEI-2010, total HBI scores (summation of all categories) range from 0 to 100, with higher scores indicating greater adherence to guidelines and overall higher beverage quality. The HBI’s preliminary validity was determined by the significant association of HBI scores with reduced cardio-metabolic health risks within a general adult population in the United States, using data from the National Health and Nutrition Examination Survey (NHANES) [16]. However, there is a need for longitudinal and/or intervention studies to examine changes in HBI scores over time, as the HBI may be useful when assessing the impact of nutrition interventions. Furthermore, causality from poor beverage consumption patterns may be determined by associating changes in HBI scores with changes in health risk factors over time.

Although dietary pattern research has been identified as a significant research gap by the US Dietary Guidelines Committees [17], minimal research has been conducted to assess what compensatory changes occur in dietary/beverage patterns when interventions focus on the reduction of singular dietary items (*i.e.*, SSB reduction) [9]. Furthermore, it has been demonstrated that healthier beverage patterns have been shown to reflect healthier overall dietary patterns; however, the majority of the available data is cross-sectional [9,18].

The Talking Health study was a six-month, community-based, two-arm randomized controlled behavioral trial with a primary focus on reducing SSB consumption (SIPsmartER group) or increasing physical activity through a matched-contact comparison group (MoveMore) [2]. Although the primary aim for the SIPsmartER group was the reduction of SSB, in order to sufficiently target SSB consumption, participants were educated on recommendations for all beverage categories (e.g., water, non-calorically sweetened beverages, and milk). Briefly, the main study findings showed a significant reduction of SSB among the SIPsmartER participants as compared to the MoveMore group at six months [19]. The objectives of the current secondary analyses are to explore the longitudinal changes in overall beverage quality (HBI) between the SIPsmartER and MoveMore participants, and to determine if changes in HBI scores (specifically SSB, total beverage energy, and total HBI score) were significantly correlated with changes in HEI-2010 scores (specifically the empty calorie component and total HEI scores). Given the significant change in SSB consumption for the SIPsmartER group demonstrated by the main trial [19], for the present analysis we hypothesized that the stated HBI component scores would significantly improve for SIPsmartER participants, and minimal to no changes would be noted for the MoveMore group. In addition, we hypothesized that changes in HBI and HEI-2010 scores would be significantly correlated, given that previous research indicated a significant relationship between beverage consumption and dietary quality [9,18]. All hypotheses were made *a priori*.

**Table 1:** Healthy Beverage Index Components [16].

Beverage Component	Description	Points
Water	Water comprises ≥20% of fluid requirements	15
	No water consumption	0
	Water is >0% but <20% of fluid requirements	Proportional
Coffee and Tea	Unsweetened coffee and tea comprise 0%–40% of fluid requirements	5
Low-fat Milk	<1.5% fat, fat-free, and/or soy milk comprises 0%–16% of fluid requirements	5
Diet Drinks	Artificially sweetened beverages comprise 0%–16% of fluid requirements	5
100% Fruit Juice	100% fruit juice comprises 0%–8% of fluid requirements	5
Alcohol	Between 0–1 drinks for women, 0–2 drinks for men	5
Full-fat Milk	0% of fluid requirements come from 2% fat or full-fat milk	5
Sugar-sweetened Beverages	Sugar-sweetened beverages are 0%–8% of fluid requirements	15
Total Beverage Energy	Energy from beverages <10% of total energy	20
	Energy from beverages ≥15% of total energy	0
	Energy from beverages is >10% but <15% of total energy	Proportional
Met Fluid Requirements	Amount of beverages (mL) consumed was greater than or equal to fluid requirements	20
	Amount of beverages (mL) consumed was less than fluid requirements	Proportional

This is the first investigation to assess changes in HBI scores over time, in response to a SSB intake reduction trial. Our findings suggest that individual HBI component scores, beyond the SSB component, are influenced by reducing SSB consumption. Furthermore, changes in total HBI scores are significantly correlated with changes in total HEI-2010 scores.

## 2. Experimental Section

### 2.1. Subjects and Study Design

Participants (*n* = 301) were recruited from medically-underserved rural regions (Medical Underservice Index score of 62 or less) [20] in southwest Virginia for this trial, known as Talking Health [2]. Talking Health was a six-month, community-based, two-arm randomized controlled behavioral trial, which targeted SSB consumption behaviors among low-socio-economic status adults, as compared with a matched-contact comparison group targeting physical activity behaviors. Guided by concepts in health literacy and the Theory of Planned Behavior, the Talking Health [2] six-month intervention period targeted SSB or physical activity behaviors through three small-group educational classes, one live teach-back call, and eleven interactive voice response phone calls. The SIPsmartER group’s primary intervention goal was to reach the recommendation of less than 8 fl oz of SSB per day [21,22], and the MoveMore group’s primary intervention goal was to reach 150 minutes of moderate-intensity aerobic activity and muscle-strengthening activities on two or more days per week [19].

Recruitment details are published elsewhere [2]; briefly, participants were recruited from April 2012 to September 2013 through various active (day care centers, festivals, health/free clinics, health departments, local extension agents) and passive (targeted mailings, flyers, radio announcements, newspaper ads) recruitment methods. To be eligible, participants had to consume at least 200 kcal/day from SSB, as assessed by the validated BEVQ-15 (a beverage intake questionnaire) [23,24,25,26], prior to enrollment. Additionally, participants had to be English-speaking adults ≥18 years old, report no physical activity limitations, and could not be currently enrolled in any other nutrition or physical activity programs. Although pregnancy status was not an exclusion criterion, women who were pregnant at baseline or became pregnant during the six-month intervention were excluded from this analysis (*n* = 5). Additionally, participants with artificial sweetener consumption greater than three standard deviations from the mean were excluded from the analysis (*n* = 4), as significant misreporting of artificial sweetener was suspected. Participants were randomized into either SIPsmartER or MoveMore after completing baseline assessments.

### 2.2. Methods

Participants underwent baseline and six-month follow up assessments of height, measured in meters without shoes using a portable stadiometer; weight, measured in light clothing without shoes, to the nearest 0.1 kg using a digital scale (model 310GS; Tanita, Tokyo, Japan); and BMI was calculated. Participants provided demographic information, and dietary intake was collected using three 24 h dietary intake recalls [27,28]. The first 24-h dietary recall was completed in-person and the two remaining dietary recalls were completed unannounced via telephone; recalls were collected by trained research technicians who were supervised by a doctoral-level registered dietitian. One weekend and two weekdays were recalled to provide a more accurate representation of habitual dietary habits. The dietary intake recalls were analyzed using the Nutrition Data System for Research (NSD-R) nutritional analysis software (Nutrition Coordinating Center, University of Minnesota, Twin Cities, MN, USA, 2011). Information regarding physical activity behaviors (minutes of moderate-vigorous physical activity and strength training) was collected through the Godin questionnaire [29].

### 2.3. Healthy Beverage Index and Healthy Eating Index

HBI and HEI-2010 scores were calculated using dietary intake recall data; specifically, food group servings, including beverages, were extracted from the NDS-R food group output files. HBI total (ranging from 0 to 100 points) and sub-component scores were calculated according to Duffey and Davy’s guidelines [16]. See Table 1 for scoring information. HEI-2010 total and sub-component scores were derived from NDS-R output files based on guidelines developed by NDS-R, and scores were calculated according to a standardized published protocol, which includes an adjustment for energy intake [15,17]. HEI-2010 scores range from 0 to 100 points (HEI total score is the sum of all twelve component scores) and the empty calorie HEI scores range from 0 to 20 points, with higher scores indicating greater adherence to the 2010 Dietary Guidelines for Americans [30].

### 2.4. Ethics

This study was conducted according to the guidelines laid down in the Declaration of Helsinki and the Virginia Tech Institutional Review Board approved the study protocol. Participants provided written informed consent prior to enrollment.

### 2.5. Statistics

For demographic characteristics, descriptive statistics (means and standard deviations; frequencies), ANOVA (F) tests (compared means across conditions), and χ^2^ tests (compared proportions across conditions) were performed using SPSS statistical software version 23 (IBM, Armonk, NY, USA, 2015). Multilevel mixed-effects linear regression analyses were performed using Stata software version 13 (StataCorp LP, Texas, TX, USA, 2013) to account for clustering of individuals within cohorts. Correlational analyses were also conducted using Stata. Results of intention-to-treat (baseline value carried forward) analyses are presented [31,32]. Individual baseline characteristics were controlled in accordance with the primary outcome paper [19], including age, gender, race/ethnicity, BMI, income, education level, health literacy level, employment status, number of children, and smoking status. The Talking Health study was powered to detect a small effect size of 0.34 for 0–6 changes in SSB intake between the SIPsmartER and MoveMore conditions (*i.e.*, 80% power, 0.05 type 1 error) [19]. The final analytical sample was 292 participants. The alpha level was set *a priori* at *p* ≤ 0.05.

## 3. Results

### 3.1. Participants

Demographic information is presented in Table 2. Briefly, participants (*n* = 292; SIPsmartER *n* = 149; MoveMore *n* = 143) were primarily female and Caucasian. The mean reported age was 42 ± 13 years old (range 18–81) and the majority of participants were considered overweight or obese (79%), with an average BMI of 33 ± 9 kg/m^2^ (range 16–72). When comparing baseline demographic characteristics between SIPsmartER and MoveMore groups, no significant differences were found (*p* > 0.05). The retention rate at the six-month data collection point was 72% (*n* = 210/292).

**Table 2:** Baseline sample characteristics by randomized condition assignment ^a^.

Characteristics	Total Sample (*n* = 292)	SIPsmartER (*n* = 149)	MoveMore (*n* = 143)
Age (years), M ^b^ (SD) ^c^	42.0 (13.4)	41.8 (13.4)	42.3 (13.4)
Gender			
Male	55 (19)	30 (20)	25 (17.5)
Female	237 (81)	119 (80)	118 (82.5)
Race			
Caucasian	271 (93)	135 (90.5)	136 (95)
African American	13 (4.5)	10 (7)	3 (2)
More than one race	7 (2.0)	3 (2)	4 (3)
Other	1 (0.5)	1 (0.5)	0 (0)
Ethnicity			
Hispanic/Latina	3 (1)	2 (1)	1 (0.5)
Education Level			
≤High school graduate	90 (31)	48 (32)	42 (29.5)
Some college or greater	202 (69)	101 (68)	101 (70.5)
Anthropometry			
Weight (kg), M (SD)	90.6 (25.4)	90.5 (26.4)	90.6 (24.4)
BMI (kg/m^2^), M (SD)	33.0 (9.1)	33.2 (9.3)	32.8 (9.0)
Underweight (≤18.4)	5 (1.5)	3 (2)	2 (1.5)
Normal weight (18.5–24.9)	58 (20)	28 (19)	30 (21)
Overweight (25–29.9)	63 (21.5)	34 (23)	29 (20.5)
Obese (≥30)	166 (57)	86 (56)	82 (57)
Obese class 1 (30–34.9)	59 (20)	29 (19.5)	30 (21)
Obese class 2 (35–39.9)	43 (15)	20 (13)	23 (16)
Obese class 3 (≥40)	64 (22)	35 (23.5)	29 (20)

^a^ *n* (%) unless otherwise noted; ^b^ M = Mean; ^c^ SD = Standard Deviation.

### 3.2. Beverage Quality

As hypothesized, overall beverage quality (total HBI score) significantly increased during the six-month intervention period for the SIPsmartER participants by 7.5 points (Table 3). Additional changes in individual HBI components for the SIPsmartER group included significantly improved SSB and total beverage calorie HBI component scores. However, the low-fat milk and diet beverage components significantly decreased with these improvements. Although the MoveMore group also significantly improved total HBI scores (*p* ≤ 0.001), a significant between group effect was present (*p* ≤ 0.05) and the noted increase was less than half of the change demonstrated by the SIPsmartER participants. The significant contributors to the increase in total HBI scores for MoveMore participants were water, whole milk, and total beverage calories; the SSB HBI component did not significantly improve for the MoveMore participants.

**Table 3:** Changes in Healthy Beverage Index scores from baseline to 6-month by treatment group using an intention-to-treat analysis ^a^.

Healthy Beverage Index Variable (Possible Score)	Group	Baseline ^b^	6-Month ^c^	Adjusted Change Baseline to 6-Month ^c,d^	*p*-Value Group by Time
Water (0–15)	SIPsmartER ^e^	10.4 (6.0)	11.0 (6.1)	0.6 (−0.3, 1.5)	NS ^e^
	MoveMore ^e^	11.0 (5.8)	12.1 (5.5)	1.1 * (0.2, 2.1)	
Tea and Coffee (0–5)	SIPsmartER	4.8 (1.0)	4.4 (1.6)	−0.4 (−0.8, 0.1)	NS
	MoveMore	4.8 (1.1)	4.7 (1.2)	−0.1 (−0.3, 0.2)	
Low-fat Milk (0–5)	SIPsmartER	4.8 (0.9)	4.6 (1.4)	−0.2 *** (−0.4, −0.1)	≤0.05
	MoveMore	4.6 (1.3)	4.8 (1.1)	0.1 (−0.2, 0.4)	
Diet Soda (0–5)	SIPsmartER	4.3 (1.8)	3.8 (2.1)	−0.4 * (−0.9, 0.0)	≤0.05
	MoveMore	4.4 (1.7)	4.4 (1.6)	0.0 (−0.1, 0.2)	
100% Fruit Juice (0–5)	SIPsmartER	4.8 (1.0)	4.7 (1.2)	−0.1 (−0.3, 0.1)	NS
	MoveMore	4.6 (1.4)	4.5 (1.5)	−0.1 (−0.3, 0.2)	
Alcohol (0–5)	SIPsmartER	4.8 (1.0)	4.9 (0.8)	0.1 (0.0, 0.2)	NS
	MoveMore	4.8 (1.1)	4.8 (1.0)	0.0 (−0.1, 0.2)	
Whole Milk (0–5)	SIPsmartER	4.4 (1.6)	4.7 (1.1)	0.3 (−0.1, 0.8)	NS
	MoveMore	4.3 (1.8)	4.7 (1.2)	0.4 *** (0.2, 0.6)	
Sugar-sweetened Beverages (0–15)	SIPsmartER	1.3 (4.3)	4.2 (6.8)	2.9 *** (1.8, 4.0)	≤0.01
	MoveMore	2.0 (5.1)	2.5 (5.6)	0.5 (−0.5, 1.6)	
Total Beverage Calories (0–20)	SIPsmartER	4.4 (7.5)	8.6 (9.4)	4.2 *** (2.6, 5.8)	≤0.05
	MoveMore	4.6 (7.6)	6.2 (8.3)	1.6 *** (0.8, 2.4)	
Total Fluid Consumption (0–20)	SIPsmartER	16.5 (4.4)	17.1 (4.2)	0.6 (−0.2, 1.3)	NS
	MoveMore	16.7 (4.2)	17.4 (4.0)	0.7 (−0.3, 1.6)	
Total HBI Score (0–100)	SIPsmartER	60.4 (13.4)	67.9 (17.9)	7.5 *** (5.4, 9.7)	≤0.05
	MoveMore	61.6 (14.6)	64.9 (16.4)	3.4 *** (1.6, 5.2)	

^a^ Intention-to-treat uses baseline observation carried forward imputations procedure, *n* = 292; ^b^ Means (Standard Deviations) are not adjusted for covariates; ^c^ Models are controlled for baseline covariates including age, gender, race/ethnicity, BMI, income, education level, health literacy level, employment status, number of children, and smoking status. Change scores and 95% confidence intervals are adjusted for covariates; ^d^ Within group statistical significance indicated by bold face asterisks: * *p* ≤ 0.05; *** *p* ≤ 0.001; ^e^ SIPsmartER condition (*n* = 149); MoveMore condition (*n* = 143); NS = not significant.

A significant decrease in BMI for SIPsmartER participants was demonstrated (−0.21 kg/m^2^, *p* < 0.01), which substantiated the validity of improved beverage consumption patterns, as compared to a non-significant increase in BMI for MoveMore participants (0.10 kg/m^2^, *p* > 0.05) [19]. However, no significant correlation was observed between the total HBI score and BMI (*r* = −0.09, *p* = 0.13) over the intervention period.

### 3.3. Dietary Quality *versus* Beverage Quality

As hypothesized, components of the HBI and HEI were significantly correlated. Specifically, the SSB and total beverage calorie HBI components, as well as the total HBI score, were significantly correlated with the empty calorie HEI score. Additionally, the SSB HBI component and the total HBI score were significantly correlated with total HEI scores (Table 4).

**Table 4:** Correlation of changes between Healthy Eating Index and Healthy Beverage Index components.

Variables	Empty Calorie: Healthy Eating Index-2010	Total Healthy Eating Index-2010
Sugar-sweetened Beverage: Healthy Beverage Index	0.32 ***	0.20 **
Total Beverage Calories: Healthy Beverage Index	0.29 ***	0.10
Total Healthy Beverage Index	0.27 ***	0.15 **

** *p* ≤ 0.001; *** *p* ≤ 0.0001.

### 3.4. Physical Activity and Beverage Quality

A mean increase of 15 min (*p* ≤ 0.01) for MoveMore *versus* an increase of 3 min of moderate-vigorous physical activity (*p* > 0.05) for SIPsmartER and mean increase of 17 min (*p* ≤ 0.01) for MoveMore *versus* a mean decrease of 3 min of strength training physical activity (*p* > 0.05) for SIPsmartER was demonstrated [19]. However, after assessing the change correlations between the HBI (SSB, water, fluid requirement, and total score) and physical activity (minutes of moderate-vigorous activity and strength training), no significant associations were found (*p* > 0.05), with the exception of a negative association between the HBI SSB component and minutes of strength training (*r* = −0.14, *p* = 0.02).

## 4. Discussion

This was the first investigation to assess longitudinal changes in overall beverage intake quality in response to a SSB intake reduction intervention. Our findings suggest that individual HBI component scores for the SIPsmartER participants, beyond the SSB component, were influenced by intervention strategies that primarily focused on SSB reduction. Furthermore, changes in total HBI scores were significantly correlated with changes in total HEI-2010 scores.

The SSB and total beverage calorie components of the HBI, as well as the total HBI score, significantly increased for the SIPsmartER group. Improvement in the total HBI score for SIPsmartER was threefold the increase demonstrated by the SSB component of the HBI.

Even though the SSB HBI score for the SIPsmartER group significantly improved after the intervention, participants were still only able to achieve a score of 4.2 out of 15. This may be attributed to SSB outcome goals differing between Talking Health (less than 8 fl oz/day) [2] and the HBI (less than 8% of fluid requirements) [16]. On average, participants consumed 1700 kcal/day [32], which translated to a fluid recommendation of 1700 mL/day (1 mL/kcal). Thus, to achieve a maximum score of 15, participants had to consume less than 4.5 fl oz of SSB per day. Although SIPsmartER participants significantly decreased their SSB fl oz intake by 50% [19], their intake would have needed to decrease by approximately 20 additional fl oz in order to attain a score of 15.

As shown in Table 3, with the increase in SSB and total beverage calorie HBI components, there was a concurrent decrease in low-fat milk and diet soda scores. This caused a shift in the beverage pattern beyond reducing SSB consumption; however, no displacement in the total fluid component was noted, even with the improved total beverage calorie HBI score. Thus, it was theorized that SSB beverages were replaced with other alternatives, such as diet soda. Although artificially-sweetened beverages did not significantly increase for the SIPsmartER group during the intervention, the between group effect was found to be significant (*p* ≤ 0.05) [32]. However, as diet soda can only comprise up to 16% of the fluid requirement (equivalent to 9 fl oz for this sample), this may have caused the diet soda HBI score to significantly decrease as a single can of diet soda equals 12 fl oz. An increase in water consumption could have attenuated this effect, however water HBI scores did not improve for the SIPsmartER participants.

**Figure 1:**
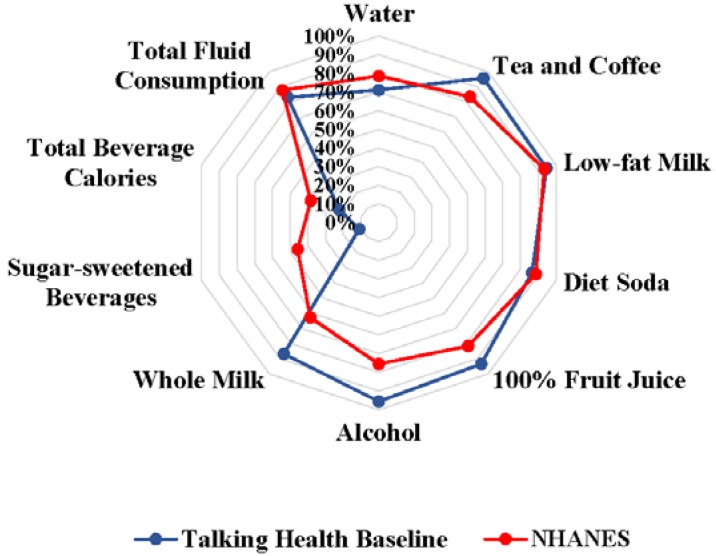
Graphical representation of Healthy Beverage Index component scores from NHANES as compared to baseline Talking Health participant values. Score of 100% for each Healthy Beverage Index component represents achieving the maximum number of points for each Healthy Beverage Index component. NHANES, National Health and Nutrition Examination Survey (2005–2010).

As previously mentioned, this is a novel area of research, to which there are no comparable studies that assess changes in HBI over time. However, the primary manuscript for the HBI [16], which included HBI values derived from the National Health and Nutrition Examination Survey (NHANES) 2005–2010 (*N* = 16,252), can be used for comparison of this sample’s baseline HBI scores to nationally representative values. As depicted in Figure 1, Talking Health and NHANES HBI component scores exhibited similar patterns, with the exception of Talking Health participants having lower SSB and total beverage calorie HBI components. This was a plausible deviation from expected values as the targeted intervention population had to consume a minimum of 200 calories per day from SSB to be eligible to participate. Regardless of these deviations, total fluid consumption was comparable between the two samples. The total HBI score for Talking Health participants at baseline was 8 points lower than values obtained from NHANES data; however, after the six month intervention, SIPsmartER participants’ total HBI scores increased to match national HBI scores of 68 points [16]. A 10 point increase in total HBI score is thought to be a clinically significant change, as many reduced risks in cardio-metabolic outcomes were observed with an increase of 10 points [16]. As participants only experienced an increase of 7.5 points, this may explain the lack of a significant correlation between the HBI score and BMI.

Our previous work demonstrated that changes in dietary intake (via HEI-2010) were concurrent to a reduction in SSB consumption [32]. Our current findings produced a similar effect, in that changes in HBI were significantly correlated with changes in HEI-2010 in response to the SSB intervention. Furthermore, as the HBI is able to overcome the limitations of the HEI-2010 for beverage quality assessment, and healthier beverage patterns have been shown to represent healthier dietary patterns, the HBI may be able to provide diet quality information beyond the HEI-2010 by specifically assessing the total intake of calories from beverages and the ability to meet fluid recommendations, which is important for overall health [4,33].

Contrary to our hypothesis, several HBI components (water, whole milk, and total beverage calories), as well as the total HBI score, significantly improved for the MoveMore group. This may be attributed to the participants’ exposure to behavioral and theoretical questions related to consumption of all beverages (SSB, water, diet soda, *etc.*) during the outcome data collection points. As the MoveMore participants significantly increased their physical activity as compared to the SIPsmartER participants, it was theorized that the improvement in HBI water scores may have been attributed to increased physical activity; however, no positive associations were demonstrated between physical activity and HBI scores. An additional contribution to improved HBI scores may have been due to spontaneous changes in beverage consumption in response to beginning a physical activity regimen. This effect was demonstrated by Halliday *et al.*, where upon enrolling in a physical activity intervention, with no dietary counseling component, participants spontaneously decreased their consumption of added sugars, sweets/desserts, and other food groups [31]. Even though the MoveMore group experienced this shift in beverage consumption, overall beverage quality only improved by half the amount of SIPsmartER participants.

We acknowledge several limitations of this investigation. The first is the reliance on self-reported dietary data, which is subject to reporting error and participant bias [28]. To help offset the potential bias, we utilized gold-standard dietary recall methodology and state-of the-art nutritional analysis (NDS-R) software. Additionally, the results of this study may be not be generalizable beyond the targeted rural area of southwest Virginia; however, beverage patterns were found to be reasonably consistent with national surveillance data.

## 5. Conclusions

In conclusion, intervening upon sugar-sweetened beverage consumption led to additional improvements in individual and total HBI component scores beyond the SSB HBI component in this intervention focused on beverage intake. Furthermore, the Healthy Beverage Index may be a valid proxy of overall dietary intake and offer added benefits beyond the Healthy Eating Index when assessing dietary quality and health status.

## Abbreviations

HBI, Healthy Beverage Index

HEI-2010, Healthy Eating Index, 2010

SSB, Sugar-sweetened beverages
